# Changes in Community Structure of Resting State Functional Connectivity in Unipolar Depression

**DOI:** 10.1371/journal.pone.0041282

**Published:** 2012-08-20

**Authors:** Anton Lord, Dorothea Horn, Michael Breakspear, Martin Walter

**Affiliations:** 1 Division of Mental Health Research, Queensland Institute of Medical Research, Brisbane, Queensland, Australia; 2 University of Queensland, St Lucia, Queensland, Australia; 3 Clinical Affective Neuroimaging Laboratory, Leibnitz Institute for Neurobiology, Magdeburg, Germany; 4 Departments of Psychiatry and Neurology, Otto v. Guericke University, Magdeburg, Germany; 5 School of Psychiatry, University of New South Wales, New South Wales, Sydney, Australia; 6 The Black Dog Institute, Sydney, New South Wales, Australia; 7 The Royal Brisbane and Womans Hospital, Brisbane, Queensland, Australia; 8 Center for Behavioral and Brain Sciences, Magdeburg, Germany; Hangzhou Normal University, China

## Abstract

Major depression is a prevalent disorder that imposes a significant burden on society, yet objective laboratory-style tests to assist in diagnosis are lacking. We employed network-based analyses of “resting state” functional neuroimaging data to ascertain group differences in the endogenous cortical activity between healthy and depressed subjects.

We additionally sought to use machine learning techniques to explore the ability of these network-based measures of resting state activity to provide diagnostic information for depression. Resting state fMRI data were acquired from twenty two depressed outpatients and twenty two healthy subjects matched for age and gender. These data were anatomically parcellated and functional connectivity matrices were then derived using the linear correlations between the BOLD signal fluctuations of all pairs of cortical and subcortical regions.

We characterised the hierarchical organization of these matrices using network-based matrics, with an emphasis on their mid-scale “modularity” arrangement. Whilst whole brain measures of organization did not differ between groups, a significant rearrangement of their community structure was observed. Furthermore we were able to classify individuals with a high level of accuracy using a support vector machine, primarily through the use of a modularity-based metric known as the participation index.

In conclusion, the application of machine learning techniques to features of resting state fMRI network activity shows promising potential to assist in the diagnosis of major depression, now suggesting the need for validation in independent data sets.

## Introduction

The Major depressive disorder (MDD) is a complex disease associated with high rates of misdiagnosis [1]. The limitations of the current symptom-based approach are well recognised [2] and remain the subject of scientific debate (e.g. see [3], [4]). For example primary care physicians in the outpatients setting recognize only some 35% cases of depression [1], [5] and falsely diagnose almost 20% of patients who are not depressed [1]. Until recently, the ‘gold standard’ for assessing depression was the Hamilton scale for depression (HAM-D). However, recent evidence suggests that the HAM-D may be psychometrically and conceptually flawed [6], arguing that a fundamental reassessment of this gold standard is needed. Whereas diagnosis has traditionally focused on clinical interview and clinician or patient rating scales, recent advances in brain imaging techniques strengthen the possibility of using the structural, functional and biochemical architecture of the brain toward this goal. These advances have occurred in both the hardware and software domains, leading to higher spatial and temporal resolution, improved signal to noise ratio, advanced multivariate analysis algorithms and new data modalities such as diffusion imaging (DTI). The objective of the current paper is to leverage methodological advances towards a diagnostically informative brain imaging protocol in MDD.

Functional brain imaging has traditionally focused on task-related disturbances in key regions of interest including regions of pr efrontal and cingulate cortex, hippocampus, striatum, amygdala, and thalamus [7], [8]. Despite vigorous re search in this field, there do remain apparently disparate findings, such as up- [9], [10] versus down-regulation [11], [12] in prefrontal cortex, possibly due to task subtleties, illness sub-types and other contextual differences. A growing school of thought, however, suggests that depression is unlikely to reflect local changes in specific areas, but rather alterations in distributed network activity across large cortical and subcortical circuits [13], [14] with recent interest focussing on the default mode network [15], [16], in turn reflecting a broader paradigm shift in neuroimaging science [17]. Subjects are not required to fulfil specific (often comple x and challenging) tasks, making resting state data especially attractive for the use with patient populations.Recently [18] used whole brain resting state fMRI to identify a decrease in functional connectivity within medication-free subjects suffering accute depression. Furthermore, [19] identified the potential to use support vector machines to accurately classify depression from functional connectivity extracted from fMRI. Research has also shifted from a focus on neuronal activity evoked by affective or cognitive tasks, toward the analysis of spontaneous, ongoing “resting state” fluctuations [20].

In this paper, we use data-driven estimates of resting state “functional connectivity” (statistical correlations between spatially distinct BOLD fluctuations) to study activity-dependent network structure in MDD. Changes of resting state functional connectivity (rsFC) in MDD have previously been described in the mood regulating cortico-limbic circuit [21]–[23] - which was first described by [24] - and for the default mode network (DMN) [13], [25] - a constellation of areas that typically show greater activity at rest than during cognitive tasks [26].

One challenge facing the use of functional connectivity in between-group analyses is that a network of 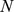 nodes (brain regions), yields 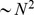 edges for possible pair-wise comparison. Although statistical methods have been developed to control the multiple comparison problem associated with such multiple data features [27], two computational techniques allow an alternative approach. The first is to study the overall topological properties of the whole-brain network, leveraging the tools of graph theory [28] to reduce the network structure to a few summary measures such as its path length (the average number of edges between all pairs of nodes), clustering (tendency of nodes to be form small cliques) and modularity (the emergence of communities of densely connected network nodes). Graph analysis of fMRI and EEG data has revealed altered network topology in a number of neuropsychiatric disorders such as Alzheimer's disease [29], ADHD [30] and schizophrenia [31], [32]. Recently, changes in network structure were identified in resting state fMRI data in patients with MDD [33]. Three findings were reported: Firstly, whole brain topological metrics showed significant between group differences, specifically the small world index (SWI) and typical path length. Secondly, using a recent method for detecting changes in the edge weights of a graph after correcting for multiple comparisons [34], differences in the functional connectivity of a sub-network of cortical regions were observed. Thirdly, a number of node-wise measures of topological organization were reported, although these were not corrected for type II error. This final question hence requires further investigation.

A second promising solution to manage the large number of network edges is to exploit advances in machine learning, such as support vector machines (SVM) and enter the entire multivariate structure into an algorithm that is capable of identifying those features that most informatively predict group membership. SVM have been shown to be useful in predicting disease classification from neuroimaging data [35] and, in contrast to traditional between group analyses of variance, have the advantage in allowing the classification of individual data sets. For example, [36] used an SVM to identify individuals who were likely to progress from an ultra high risk group to psychosis. In a recent intriguing study of MDD, [37] achieved a high level of disease classification using SVM trained on the functional connections amongst a network of 15 key regions of interest. However, the lack of close matching of ages in the clinical and control groups, together with the known effect of cortical matura tion on functional connectivity [38], again underlines the need for further research in this field.

The aim of the present study is to combine the graph theoretical analysis of resting state fMRI with machine learning techniques to pursue two objectives: (1) To predict clinical status using network measures of functional connectivity, and (2) Identify those network features that are most diagnostically informative. Although we calculated a broad suite of graph theoretical measures, we focus on those related to the community structure of the network as it has been recently identified as a sensitive marker for organization in brain networks [39]. The most salient of these is the participation index (PI) which is low for nodes embedded within local communities and high for those which connect different modules. We believe this is due to the more subtle nature of depression as opposed to schizophrenia, which has been shown to have graph metric alterations at a global scale [32]. We first report between group analyses of the participation index, using a false discovery rate to control for repeated measures. We then enter the entire suite of network metrics into a multivariate classifier to investigate which aspects of functional network structure are most informative for clinical diagnosis, hence comparing PI to a range of more frequently used measures.

## Methods

### Subjects

Twenty-two subjects with an acute MDD episode were recruited from the inpatient and outpatient department of psychiatry at the University of Magdeburg. Diagnosis was confirmed by a structured interview by a trained clinician, according to the ICD-10 criteria [40]. Four of the subjects were experiencing their first depressive episode, while the remainder consisted of recurrent MDD patients with a mean disease onset approximately 6 years prior to the study. The exact number of previous depressive episodes is not available for all patients. The length of the current episode was between one and twelve months, with a mean duration of 4.8 months. Due to the high level of misdiagnosis in the community, clinical staff with a high level of expertise in diagnosing mental health disorders performed the diagnosis of all subjects. Exclusion criteria were major medical illness, history of seizures, medication with glutamate modulating drugs (ketamine, riluzole, etc.) or benzodiazepines, prior electroconvulsive therapy (ECT) treatments and pregnancy, as well as all contraindications against MRI. Specific psychiatric exclusion criteria consisted of atypical forms of depression, any additional psychiatric disorder, and a history of substance abuse or dependence. All patients were rated by the Hamilton depression scale (Mean score 15.75, SD 4.84). Twenty-two healthy subjects without any psychiatric, neurological, or medical illness were self-referred from study advertisements. All volunteers completed the mini-international neuropsychiatric interview (MINI) to ensure the absence of any ICD-10 psychiatric disorders [41]. The study was approved by the institutional review board of the University of Magdeburg and all subjects gave written informed consent before inclusion. All subjects underwent an identical fMRI paradigm. All patients were medicated using SSRI, NRI, and SNRI alone or with new generation antidepressants including agomelatine or lithium. The composition of the sex- and age-matched groups after exclusion of one depressed female subject because of extensive head movement is described in Table 1. There was no significant difference in age (t-test, 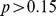) or gender between the control and clinical groups.

**Table 1:** Subject cohort.

	Healthy Controls	Depressive Patients
Number	22	21
Male/Female	13/9	13/8
Mean Age	34.55	37.86
Standard Deviation (SD) Age	6.16	11.37
HAM-D (SD)	-	15.8 (+−4.8)

### Data acquisition

The functional Magnetic Resonance imaging (fMRI) data were acquired on a 3 Tesla Siemens MAGNETOM Trio scanner (Siemens, Erlangen, Germany) with an eight-channel phased-array head coil. For acquisition of the resting-state fMRI data, the subjects were told to lie still in the scanner with their eyes closed. Functional time series of 488 time points were acquired with an echo-planar imaging sequence. The following acquisition parameters were used: echo time = 25 ms, field of view = 22 cm, acquisition matrix = 44 44, isometric voxel size = 5 5 5 mm^3^. Twenty-six contiguous axial slices covered the entire brain with a repetition time of 1250 ms (flip angle = 70). The first five acquisitions were discarded to reach steady state and limit T1 effects. High resolution T1-weighted structural MRI scans of the brain were acquired for structural reference using a 3D-MPRAGE sequence (TE = 4.77 ms, TR = 2500 ms, T1 = 1100 ms, flip angle = 7, bandwidth = 140 Hz/pixel, acquisition matrix = 256 256 192, isometric voxel size = 1.0 mm3).

### Data Preprocessing

Functional data were corrected for differences in slice acquisition time, motion-corrected using a least squares approach and a six-parameter (rigid body) linear transformation and spatially normalized [42]. The dataset of one female patient was excluded because of excessive head movement. The data were linearly detrended. An additional regression of nuisance covariates was applied during which the functional data was corrected for global mean signal as well as for white matter and cerebrospinal fluid signal. Data were preprocessed using spm5 (Wellcome Trust Center for Neuroimaging, London, England) as executed in the processing assistant for resting-state fMRI (DPARSF, [43]).

The resulting volumes were parcellated into 95 nodes using a modified version of the automatic anatomic labeling (AAL) atlas [44] containing a higher level of parcellation for the cingulate cortex [45]–[47] and insular cortex (anterior and posterior insula [48]). In contrast to the original AAL template where the cingulate regions are separate for left and right hemisphere, we combined both sides to finally obtain regions that stretch across the midline of the brain. This customization allowing co-registration with MRS voxels of interest (to be reported later) leads to 95 instead of originally 90 regions of interest (ROI). To compute the resting state functional connectivity (rsFC) of the ROIs, the fMRI time course of every ROI was extracted and Pearsons correlation coefficient was calculated pair-wise for all pairs of ROI's. The correlation coefficient reflects zero-lag undirected statistical relations of the BOLD time courses in the specific ROIs. Correlation coefficients close to 1 imply that the time courses are nearly identical, whereas those near 0 are nearly uncorrelated.

### Graph Theoretical Analysis

**Figure 1:**
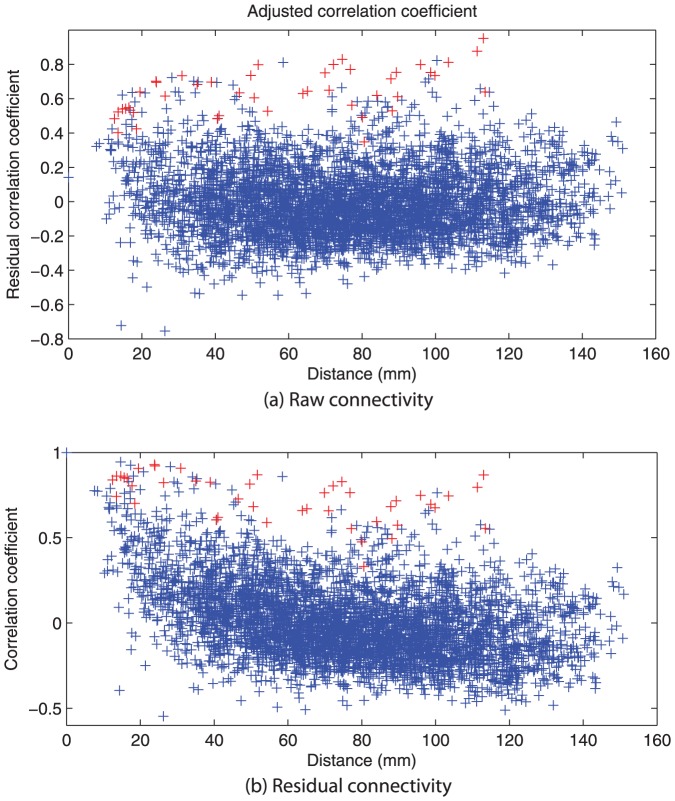
Correlation coefficients (a) uncorrected and (b) corrected for distance by the distance penalty equation (Appendix S1 A). Entries marked in red denote bilateral connections between the same region, e.g. Hippocampus left and right.

Resting state functional connectivity graphs were created by correlating the time series of all regions pair-wise in a 95 by 95 matrix (figure 1a). As in [49], these correlations show a monotonic reduction in magnitude as a function of inter-areal distance, trending toward a mean of zero, although with considerable variance (figure 1a). As this (approximately logarithmic) background trend reflects non-specific neuronal correlations between spatially adjacent neuronal populations [50], we choose to adjust the correlations as a function of distance (figure 1b), effectively regressing out these background effects and emphasising those particular correlations that are strong in magnitude relative to their spatial proximity (see Discussion for further consideration of this step). There were no significant differences in the residual errors after adjusting for distance between groups (p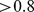). For example, this emphasises homologous inter-hemispheric pairs and other long range connections over numerous shorts-range connections between spatially proximate - although functionally unrelated - brain regions. The mathematical details of this regression are provided in Appendix S1 A.

Community structure, the focus of the current report, was determined according to the algorithm of [51]. In contrast to the other graph metrics (see below), this technique does NOT require thresholding because it does not depend on the presence or absence of connections, but rather seeks an arrangement of node's into large modules such that positive weights lie within each module and the negative weights determine module boundaries. The participation index (PI) is a feature of each nodes connectivity relative to the modularity decomposition of the entire network: Nodes with a low PI share connections with other members of the same module, whereas those with a high PI serve as connectors between modules. In all subsequent analysis, the PI scores have been rank-ordered from lowest to highest PI within each individual to remove intersubject variance. That is, a node with the lowest PI is assigned as rank 1 whilst that with the highest PI (the strongest “connector”) is ranked 95.

The other graph-theoretical metrics require the underlying globally connected networks to be rendered sparse through thresholding. Graphs were hence reduced to a sparse matrix by recursively removing edges, starting with the weakest weights and progressing unt il only 12% of edges remained. No negative connections were retained after thresholding. Any edge that would cause the graph to disconnect by its removal was retained, even in the case of a low weight. Thresholding was performed at 12% based on prior analysis that found that such sparsity was close to optimal in terms or retaining the most informative network edges whilst ensuring that disconnection is rare. Networks with fewer edges tend to have multiple disconnections (unless many bridging edges are retained), whereas the inclusion of more edges tends to introduce weaker, noisy effects obscuring between-group effects [32]. We hence derived the following network metrics from these sparse, weighted, non-directed graphs: Path length (PL) which captures the average distance between all possible pairs of nodes; Betweenness centrality (BC) which is a measure of the number of shortest paths that traverse a given node; Clustering coefficient (CC) which reflects the tendency of nodes to form local cliques, small w orld index which measures the ratio between CC and PL, and local and global efficiency (LE and GE) which captures the information capacity of the network considered as a distributed, parallel system [52].

Together these metrics capture the basic topological features of the whole brain functional connectivity. In order to control for any putative differences in overall connection strength, each metric was normalized (subject-wise) to reference random graphs. All metrics were calculated using the graph theoretical toolbox [53] implemented in MATLAB. Mathematical details are provided in Appendix S1 B.

### Statistical analysis

The PI of a given node is dependent on its connectivity and the modularity decomposition of the entire network. The modularity algorithm produces a goodness of fit score (Q) for every possible community structure. There are, in fact, a multitude of modular decompositions for each subject with near optimal goodness of fit (Q) scores. For a single subject, it is sufficient to find the single decomposition with the highest Q score and calculate the node-wise PIs using this structure. At a group level, however, these decompositions inevitably differ considerably in structure (and even number of modules) from subject to subject, presenting a challenge for group analyses. We addressed two distinct questions using the modularity structure: Firstly, are there any group differences in the PI of individual nodes for clinical versus control subjects; Secondly, can we identify group membership of clinical versus control individuals based on all graph metrics, and how does the PI contribute to this. We hence adopted two distinct, pragmatic solutions to the variability of modular structure for each of these problems.

We constrained the first question (between group analyses of node-wise PI), by ensuring that the modularity decomposition is the same for all subjects (Appendix S1 C). This means that any putative between-group differences relate exclusively to the relationship of individual nodes to this structure, and not differences in the decomposition itself. This involved an iterative procedure to identify the decomposition that gave the most cohesive group modularity structure, weighted to the Q score for individual subjects (a mathematical definition of this process together with pseudo-code is provided in Appendix S1 D). Node-wise PI scores were then extracted - relative to this modular structure - in each subject and entered into traditional between group statistical comparisons. All p-values were corrected for multiple testing using a resampling based approach to control type II error [54] based on the false discovery rate by [55].

For the second technique we sought to leverage advances in machine learning algorithms to predict group membership based on node-wise graph theoretical metrics. This question does not require that all individuals have the same community structure because between group comparisons are not performed between corresponding nodes, but rather using the entire multivariate data. We hence identified, in each subject, the single decomposition with the best Q score. Node-wise PI values were then extracted using each subjects best modularity decomposition and entered into a machine learning algorithm, together with all other graph metrics, for group classification.

### Support vector classification

A linear support vector machine was trained to predict healthy/depressed group membership, identifying the subset of the features available that maximally identified correct group membership with minimal redundancy between features [56]. The objective of feature selection is to select the optimal set of features from a dataset that optimally differentiates between groups. As the number of possible features increases, an exhaustive search quickly becomes unfeasible. The minimum redundancy, maximum relevance (mRMR) algorithm [57] uses information about each features individual predictive power, as well as the amount of mutual information with other features that have been selected to define a list of the most relevant features. mRMR produces results similar in accuracy to an exhaustive search, without the increase in time cost for ordering the feature list. Due to the combination of low computational cost, as well as high accuracy, mRMR was used for feature selection, with the results compared to a list produced by recursive feature elimination (RFE) [58].

Data was randomly split into two groups, with half used for training and the other half used for testing. Random splits where the clinical/control ratio was by chance either 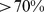 or 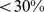 were discarded from the analysis. To improve robustness, the training/testing sets of data were reshuffled 1000 times, in a technique commonly known as bagging.

### Workflow

rsFC data is acquired from subjects in a single session. This is preprocessed using SPM5, regressing out head motion, grey matter percentage, CSF, global mean signal and parcellated into 95 nodes using a modified version of the AAL atlas. A distance dependent penalty was added to the resulting connectivity matrices and the analysis split into groupwise analysis and classification. For the groupwise analysis, a groupwise modularity structure was identified, while for the classification analysis, an individual based modular structure was selected. Graphs were thresholded and other graph metrics were calculated in the same manner for both analyses.

**Figure 2:**
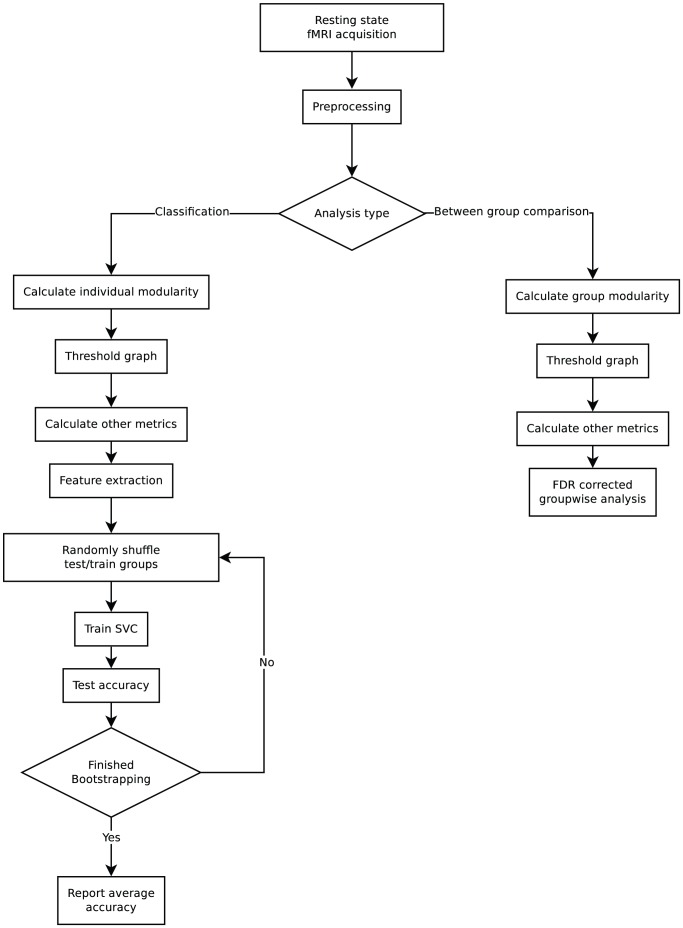
Workflow for data processing, statistical testing and machine learning in this study. All steps are described in detail in the methods section of this paper. Two analysis types were used, one for groupwise comparisons, and one for machine learning classification. The difference in modularity identification was selected so no groupwise information would influence the data used for machine learning. Thresholding and the calculation of all metrics with the exception of modularity was identical between the two analysis types.

FDR corrected p-values were calculated for all metrics and these are reported in the between group analysis section. Feature extraction was performed on all the features including the individual modularity and a support vector classifier was trained. A bootstrap approach with 1000 iterations was used to increase the robustness of the classifier and the average accuracy was reported in the classification section. This workflow has been visualised in figure 2.

## Results

### Between group analysis

**Figure 3:**
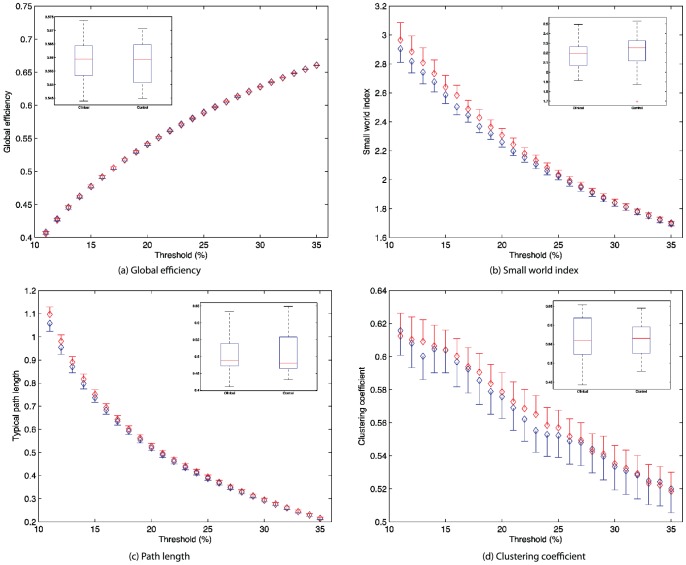
Global metrics for subjects (blue) and healthy controls (red) across a range of thresholds for connectivity sparsity from 10% to 35% in 1% increments. Inserts in each figure are the area under the curve (AUC) statistics for each metric.

We first investigated between-group effects for the traditional “whole brain” topological metrics including the clustering coefficient, path length and small world index. In order to control for any putative differences in overall connection strength, each metric was normalized (subject-wise) to reference random graphs. There were no significant differences between controls and subjects in these metrics between control and the clinical subjects, nor any suggestion of a trend effect for the path length (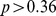), clustering coefficient (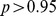) or small world index (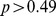) at 12% connectivity. In order to replicate the analysis of [33], we also tested global measures across a range of thresholds from 10 to 35%. No significant group differences were present at any threshold across the entire range, nor when the measures were pooled across all thresholds into a single ROC quantity (figure 3).

**Figure 4:**
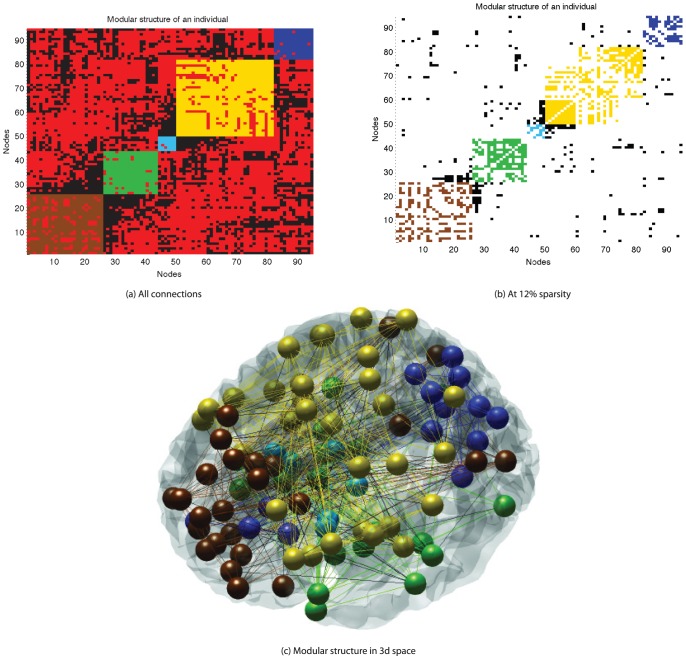
Modular structure of one healthy individual, where brown, green, cyan, yellow and dark blue represent the 5 different modules. Connections are drawn in the color of the modules if it connects nodes from the same modules, otherwise in black. Negative connections are marked in red. (a) is a fully connected graph, where correlation coefficients are modified by the distance penalty described in Appendix S1A, (b) and (c) have been thresholded to retain only 12% of edges.

We then studied the between group differences in community structure. The modularity function used has an inbuilt stopping criteria for identifying the number of modules based on both positive weights within modules and negative weights between modules. All subjects' functional networks exhibited distinct community-like structure with most modules forming bi-laterally (figure 4c). For all subjects, the optimal number of modules was either 4 or 5 (the mean number of best-fitting modules across both groups was 4.6 and the mode was 5). There was no between-group difference in this optimal number (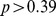). We then examined the individual goodness of fit statistics (Q-scores) as an index of how well the functional connectivity matrices show a modular structure. There were no between group differences in the Q scores (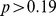). This was also true (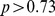) if the decomposition was confined in all subjects to have 5 modules (the most frequent number).

Previous studies have also suggested 5 modular decompositions for resting state fMRI analysis [49], [59]. We found significant similarities in modular decomposition in temporal and parietal-(pre)motor modules. We also had a large amount of overlap between the occipital and frontal modules found by [49], however in our study these two modules appeared together. One difference of particular note is in our study that the prefrontal cortex is separated from the frontal module. Included in the prefrontal module are sections of the parietal lobe. We believe the differences between our work and the earlier studies are due to the introduction of the distance dependent penalty.

**Figure 5:**
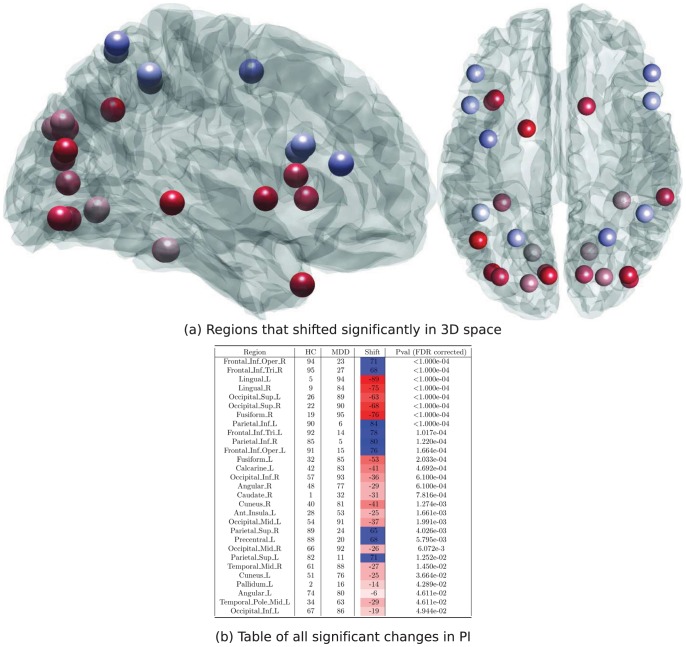
ROIs that changed in rank order between HC and MDD (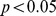, FDR corrected). Higher PI scores in the HC and MDD columns represent nodes which contain a higher porportion of connections within the module they belong to.

All further analyses was hence based on a 5 module decomposition. After adjusting for repeated measures, there were substantial differences in node-wise PI between the healthy and depressed subjects (Figure 5b). In particular 29 of the 95 nodes showed a significant difference in their participation index surviving FDR correction. Several features of these differences are noteworthy. Firstly, the shift from healthy to clinical subjects is from low PI (red) in 20 nodes, whilst to high PI (blue) in only 9 nodes. That is, nodes which tend to be deeply immersed within their local communities in healthy subjects instead frequently showed functional connections to other modules in major depression. Secondly, there was a striking spatial distribution of these effects (figure 5a). In particular, nodes whose relative PI dropped significantly in the clinical subjects (red) were distributed bilaterally through posterior and inferior regions predominantly in occipital, temporal and inferior-frontal regions. Nodes whose PI jumped in ranking in the clinical subjects (blue) were distributed through superior and anterior regions predominantly in frontal and parietal temporal regions in such a way that these two effects occur in quite distinct spatial partitions (infero-posterior versus antero-superior) of the brain.

**Figure 6:**
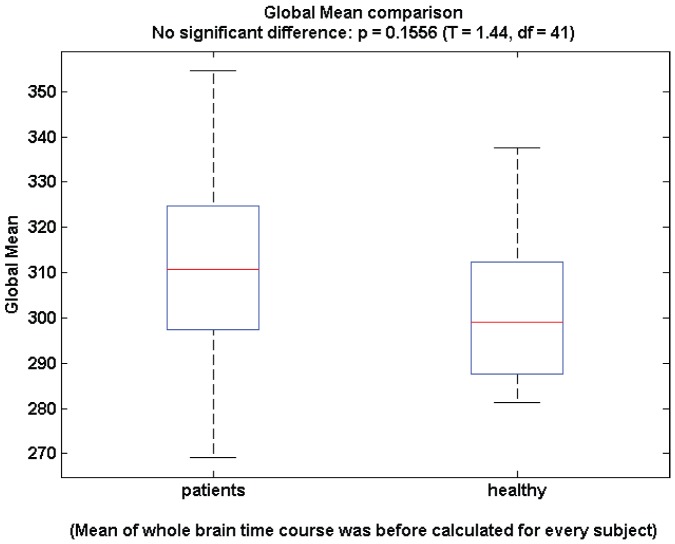
Global mean signal for healthy (left) and clinically depressed (right) subjects.

To ensure our results are do not reflect the potential confounding influence of global signal regression (a preprocessing step we employed), we undertook an additional between-group analysis of the global signal (Figure 6). There is neither a trend or a significant between group effect (p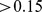).

### Classification

**Figure 7:**
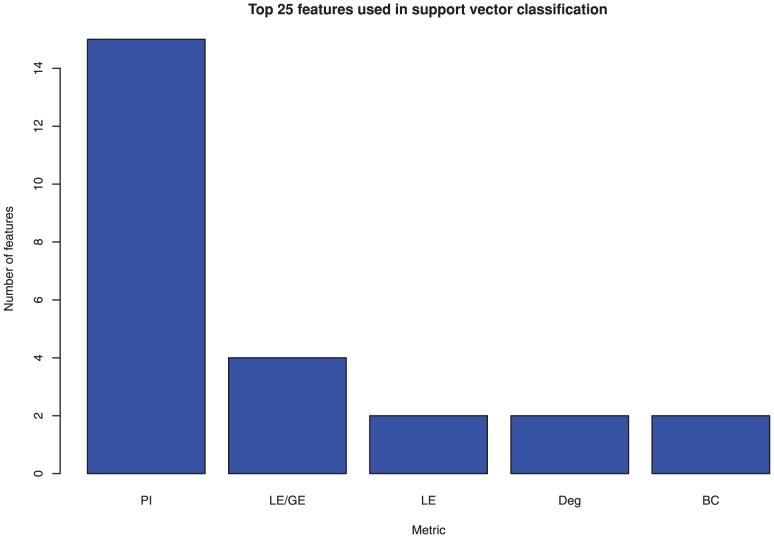
Top 25 features for classification using SVM obtained using mRMR. Metrics included are participation index (PI), local/global efficiency (LE/GE), local efficiency (LE), degree (Deg) and betweenness centrality (BC).

**Figure 8:**
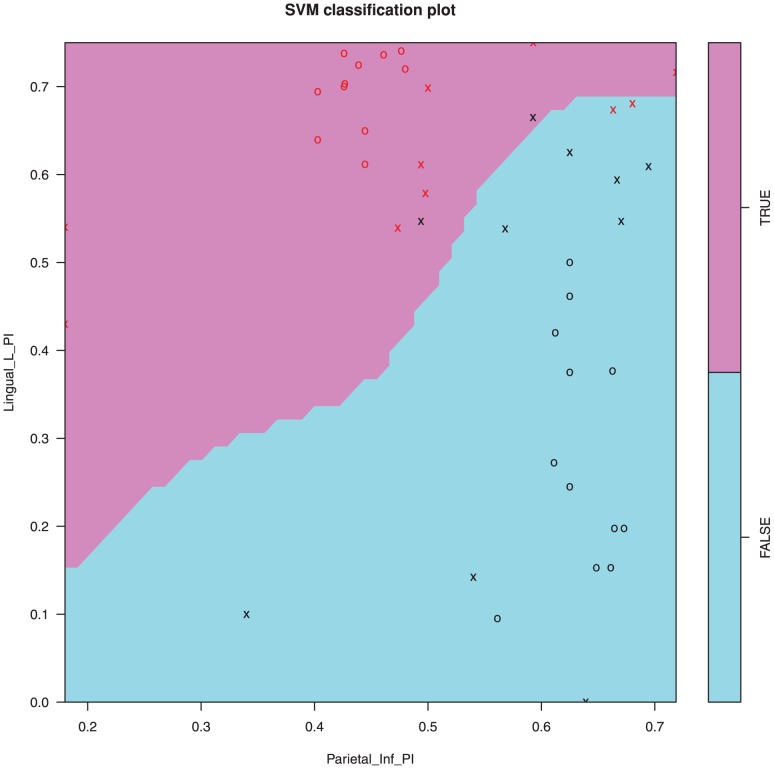
Support vector machine classification algorithm using the top two features for segregation between groups. Data points marked with an ‘x’ are used for training, while points marked as ‘o’ were used for testing.

**Figure 9:**
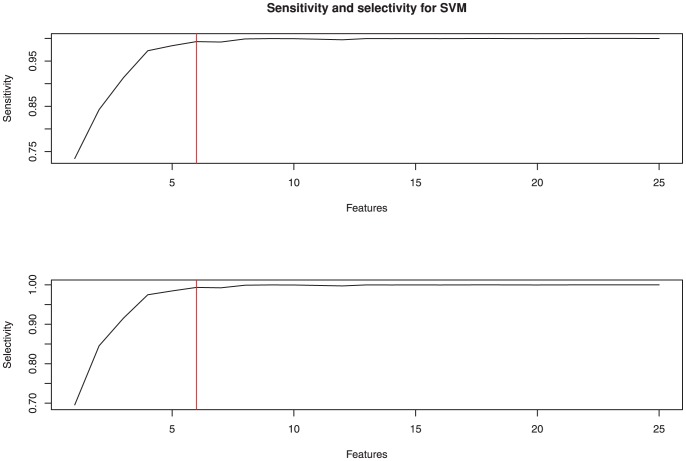
Selectivity and specificity for support vector classification using a range of features. Red line illustrates the cutoff chosen for this analysis.

The individual based modularity, which is used for the machine learning section of this paper was carried out by selecting the 5 module decomposition with the best Q value (Figure 4a) and the participation index for each node was calculated using a thresholded matrix (Figure 4b). mRMR was used to identify an optimal feature set for group classification by a support vector machine. Of the top 25 features, 15 were from the participation metric with the rest coming from degree, betweenness centrality and efficiency metrics (Figure 7). A support vector machine was trained on the best two features for illustrative purposes and shows how the two groups are separable with 90% accuracy by using only these two features (Figure 8). When the number of features used to train the SVM kernel is increased from 2 to 6, the algorithm was able to identify depressed/healthy status of individuals with an accuracy above 99% (Table 2). Only a small further improvement was hence possible by adding more than 6 features (Figure 9).

**Table 2:** Support vector machine classification accuracy.

Treatment	Test/Train	Selectivity	Sensitivity
Actual			
	Test	0.9934	0.9931
	Train	1	1
Post			
	Test	0.5263	0.5018
	Train	1	1
Pre			
	Test	0.5291	0.5016
	Train	0.5228	0.5024

Bagging results for support vector machine classification accuracy using 6 features and resampling training/testing data 1000 times. Treatment refers to the labeling of the data where ‘Pre’ and ‘Post’ refer to if the data labels were shuffled before or after vector training.

The top 25 informative features (nodes and the relevant graph metrics) are listed in table 3. As stated above, 15 of these were the PI of differing regions. The probability of any one of the graph metrics to occupy 15 or more of the top 25 data features was estimated, through resampling, to be 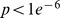, suggesting that there is a significant restructuring of the modular structure between groups.

**Table 3:** SVM features.

	Metric	Region	Side	Figure 5 pos
1	Participation Index	Lingual	Left	4
2	Participation Index	Supra marginal	Left	-
3	Degree	Parietal Inferior	Right	-
4	Participation Index	Putamen	Left	-
5	Participation Index	Frontal Inf Orb	Left	-
6	Local/Global efficiency	Thalamus	Right	-
7	Participation Index	Occipital Sup	Left	5
8	Participation Index	Frontal Inf Tri	Right	2
9	Participation Index	Parietal Inf	Right	10
10	Degree	Anterior Insula	Right	-
11	Local/Global efficiency	Rectus	Right	-
12	Participation Index	Occipital Mid	Right	22
13	Participation Index	Anterior Insula	Left	18
14	Participation Index	Precentral	Right	-
15	Participation Index	Precuneus	Left	-
16	Betweenness Centrality	Putamen	Left	-
17	Participation Index	Anterior Insula	Right	-
18	Betweenness Centrality	Anterior Insula	Right	-
19	Local/Global efficiency	Posterior MCC	Bilateral	-
20	Participation Index	Postcentral	Left	-
21	Participation Index	Frontal Inf Oper	Right	1
22	Local Efficiency	Temporal Mid	Right	24
23	Local/Global efficiency	Rostral Acc	Bilateral	-
24	Local Efficiency	Rectus	Right	-
25	Participation Index	Occipital Sup	Right	6

25 most influential features for SVC kernel.

## Discussion

The goal of the present study was to investigate topological features of resting state functional connectivity in major depression, and to explore the potential utility of using these features to predict diagnosis. In contrast to [33], we do not observe between group differences in whole brain measures of functional organization, namely path length and small world index. We do, however, observe a significant reorganization of the community structure of these networks that is a difference in the topological structure of resting state fMRI at a hierarchical level below that of the whole brain changes reported in [33]. We observed a strong pattern of increased inter modular crosstalk for superior frontal and parietal regions in MDD, while the majority of changes referred to increased within module connections of a inferior occipital parietal and subcortical set or regions. Importantly, the graph representation based on a standard anatomical template yielded a highly accurate classifier distinguishing patients and controls based on their network topology, without the need of a priorily defined regions of specific clinical interest. Participation indices in comparison to other topological measures were by far the strongest measures to contribute to group differentiation suggesting future investigations of this measure.

On the whole, we find that connector nodes in healthy subjects become locally embedded in their communities in major depression. Finally, we find that these measures provide promising diagnostic information when used in a machine learning algorithm. Just 6 measures are required to yield a very high diagnostic accuracy in our cohort. Moreover, node-wise participation indices dominate amongst all node-wise measures when ranked according to a minimum redundancy, maximum relevance algorithm.

Previous studies have shown alteration of whole brain metrics in schizophrenia (SZ) [32] and Alzheimer's disease [29]. These psychiatric disorders are associated with severe, often incapacitating disorders of cognition so it is perhaps not a surprise to find strong disturbances of global metrics in these disorders, in contrast with lack of a between group effect in our depressed cohort. Cognitive disturbances in schizophrenia are typically more significant (eg. visual/auditory hallucinations, paranoia and disorganised thinking) than in MDD where symptoms often predominate in a rather emotional domain (eg. feelings of worthlessness, helplessness and inability to experience pleasure). However, a significant between group effect in these whole brain metrics was recently reported by [33]. In order to replicate their approach, we extended our analyses across a range of thresholds from 10 to 35% for these metrics. No significant group differences were present in our data at any threshold across the entire range, nor when the measures were pooled across all thresholds into a single ROC quantity. Interestingly, the small (but non-significant) group mean differences in path length and small world index diminished as the threshold was increased, consistent with an effect of introducing increasingly noisy effects into the analysis. Given that we used the same pre-processing steps as [33], the contrasting findings may be instead due to more severely depressed subjects in their study (their clinical subjects average HAM-D scores were 24.1 compared to our 15.8). Alternatively their clinical subjects were drug naïve nature compared to our cohort of medicated subjects. It is possible that the normalizing influence of the pharmacotherapy (or the disorders natural history) was associated with a normalization in these whole brain metrics, suggesting that the intermediate modularity scale may be more sensitive to milder illness. Ethnic or cultural differences (a Chinese versus a German cohort) may also influence the classification or expression of depression between these populations and may have influenced this result.

As the objective of our paper was to investigate the potential for resting state metrics to provide diagnostically informative information, and not to focus on pathophysiology per se. At this stage it may be premature to link the list of informative data features to prior theories of brain dysfunction in depression. This is firstly because modularity decompositions are quite new to the f ield and relate to a level of organization between regional and global: There is hence no theoretical framework to make strong lin ks between prior (local and regionally focussed) studies and our approach. Importantly, functional characterisations of core regions are normally derived from direct activation studies, which then would best let us interpret abnormal task elicited responses. Here the PI would not describe the focal change in activation but rather its different implementation into functional modules, which may be highly dependent from nodal connectivity changes in other structures. Secondly, our study numbers are relatively modest such that replication in a larger, independent data set would be advisable before inferring pathophysiology.

The reorganization of community structure in MDD and its putative diagnostic use our primary findings were achieved by restricting the number of modules in all subjects to 5. Although many different modular systems of the brain have been presented recently including 6 modules [60], 5 modules [61] and 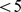 modules [62] our study shows a modular decomposition of the same order as [61] while using a modularity algorithm that utalized fully connected graphs by simultaneously maximizing positive and minimizing negative connections within modules. The main benefit of this apporach is that no thresholding of connectivity matrices is required, removing a potential confounding factor that other methods rely on. It should also be noted that many of the modularity based analyses performed so far are based on structural scans, and although there is evidence that structural and functional abnormalities can be linked [63], it is reasonable to believe that this relationship will not be exact and the resulting modules will have some variation.

Objective classification of MDD has been a goal of mental health research for some time. This has become achievable through recent technological advances in both data acquisition (fMRI) and processing (feature selection and classification algorithms) techniques. A recursive feature selection was employed by [37] to identify a set of correlations between regions (obtained by fMRI) which were then used to predict MDD using SVM. They also showed that none of their selected features for the SVM showed statistically significant differences between groups at the 95% CI (false discovery rate (FDR) adjusted). Since then, the use of graph theoretical metrics to identify meaningful measures has become widely available [53]. It has been shown [61] that significant correlations between a large number of the standard graph metrics exist. Further to this, increasing the number of features selected for a SVM to use increases the risk of over-fitting the data. Therefore a feature selection algorithm that utilises both feature relevance and mutual information is required. We utilized mRMR [57] to identify a subset which maximizes predictive power while minimizing redundant information. The use of a both well established as well as emerging graph metrics has enabled us to train a classifier with very high accuracy. This accuracy was checked by reshuffling training and testing sets 1000 times as well as randomly shuffling labels, both prior and post training: Our classifier then performed no better than random chance, arguing strongly against an over-training effect. The use of subjects who are currently receiving medication based treatment offers a sample representative of depression in the wider community. Furthermore, the diverse nature of the medication creates a more heterogeneous sample, reducing the risk of capturing a subsample of the population with a machine learning classifier. Although resting state acquisitions of EEG and fMRI have been increasing in their use recently [20], there remains active debate on the optimal preprocessing techniques which should be used. Unresolved issues in preprocessing include the influence of the parcellation method and global mean regression. We used the AAL for the basis of our parcellation atlas as it is a commonly used technique in resting state fMRI studies and is highly reproducible. However, it is known that the number of regions and their relative volume used in a parcellation scheme systematically influences the graph theoretical-derived features [34]. Recently automated fine grained parcellation techniques have started to be used instead of traditional, larger atlases [64]–[66], however no consensus has yet been reached on how to optimally generate parcellation maps. This motivated our use of the AAL atlas, with minimal modifications. Similarly recent research has shown that in simulated data, anti-correlations can be introduced through global mean regression where none previously existed [67]. However it is also well known that fMRI data are confounded by very large spatial signal fluctuations that arise from a variety of non-neuronal physiological processes (such as respiration) whose presence dominates nave correlation matrices derived without global signal regression. Moreover, because global regression can be shown to introduce anti-correlations in synthetic data it does not follow that the same do not indeed occur amongst resting state neuronal populations as shown in several computational studies [68], [69] only to become obscured by high amplitude, large scale artifacts. The idea that these negative correlations are biologically relevant was recently reiterated by [70]. The modularity algorithm employed leverages negative weighting to identify the modular structure. As strength also uses negative weighting, but did not rate highly amongst the features identified with our mRMR algorithm, it seems that the negative weights do not on their own contribute to the diagnostic accuracy. To allow comparison with most prior studies, we employed a global signal regression step. In this respect, it is important to note that there were no between group differences in the global signal of our data (Figure 6).

Head motion has also been recently recognised as a potential confounder in between group analyses of resting state connectivity [71]. We carefully inspected all data and excluded subjects with excessive head motion. All remaining subjects had a maximum of 3 mm of movement and 3 degrees of rotation. We additionally corrected for head motion prior to estimating functional connectivity. This step, as well as the regression of the other nuisance variables (CSF, white matter) have been suggested as important in reducing the influence of head motion on between group analyses [71]. Finally, the potential influence of excessive head motion is a bias towards short-range connections and away from longer distance functional connections. In fact we explicitly adjusted our data for distance-dependent effects (see Methods) mitigating against such a possible confound.

Future work will include cross-validation in larger, independent data sets and is required before the present findings could be considered for translation into the clinical use. Underlying physiological mechanisms may also be elucidated using multi-modal data and/or computational models such as network discovery for DCM [72]. The combination of the present data-driven functional graph metrics with complimentary information about structural connectivity (through the use of diffusion weighted data) and/or synaptic biochemistry (via magnetic resonance spectroscopy, MRS) would also be informative.

## Supporting Information

Appendix S1 (PDF)
