# Supplementary material for: Changes in Community Structure of Resting State Functional Connectivity in Unipolar Depression
Source: PLoS One. 2012 Aug 20;7(8):e41282. doi: 10.1371/journal.pone.0041282 (PMC3423402; doi:10.1371/journal.pone.0041282)
Supplement: Appendix S1 — (PDF) [file pone.0041282.s001.pdf]

Changes in community structure of resting state brain networks in unipolar depression

Supplementary equations

Anton Lord[1,2,*], Dorothea Horn[3,4], Michael Breakspear[1,5,6,7], Martin Walter[3,4,8]
[1]Division of Mental Health Research, Queensland Institute of Medical Research, Brisbane, QLD, Australia
[2]University of Queensland, St Lucia, QLD, 4001, Australia
[3]Clinical Affective Neuroimaging Laboratory, Leibnitz Institute for Neurobiology, Brenneckestrasse 6, 39120 Magdeburg
[4]Departments of Psychiatry and Neurology, Otto v. Guericke University, Leipziger Strasse 44, 39120 Magdeburg
[5]School of Psychiatry, University of New South Wales
[6]The Black Dog Institute, Sydney, NSW, Australia
[7]The Royal Brisbane and Womans Hospital, Brisbane, QLD, Australia
[8]Center for Behavioral and Brain Sciences (CBBS), Magdeburg, Germany
∗ **E-mail: antonL@qimr.edu.au**

Appendices

A Distance dependant penalty

$$w_{adj} = w\frac{-log\,(x) + k}{k} - r$$

B Graph metric definitions

Participation index

$$y_i = 1 - \sum_{m \in M} \left(\frac{k_i(m)}{k_i} \right)^2 \tag{1}$$

where M is the set of modules obtained by the [1] method, k_i is the number of edges connected to node i and $k_i(m)$ is the number of links originating at node i which finish within module m.

Characteristic path length

Characteristic path length of the network [2].

$$L^w \frac{1}{n} \sum_{i \in n} \frac{\sum_{j \in N, j \neq i} d^w_{ij}}{n-1} \tag{2}$$

Local efficiency

Local efficiency of the network [3].

$$E^w_{loc} = \frac{1}{2} \sum_{i \in N} \frac{\sum_{j,h \in N, j \neq i} \left(w_{ij} w_{ih} \left[d^w_{jh} \left(N_i \right) \right]^{-1} \right)^{\frac{1}{3}}}{k_i \left(k_i - 1 \right)} \tag{3}$$

Global efficiency

Global efficiency of the network [3].

$$E = \frac{1}{n} \sum_{i \in N} \frac{\sum_{j \in N, j \neq i} \left(d_{ij}^{w}\right)^{-1}}{n-1} \tag{4}$$

Clustering coefficient

Clustering coefficient of the network [2].

$$C = \frac{1}{n} \sum_{i \in N} \frac{2t_i^w}{k_i \left(k_i - 1\right)} \tag{5}$$

Betweenness centrality

Betweenness centrality of node i [4].

$$b_i = \frac{1}{\left(n-1\right)\left(n-2\right)} \sum_{h,j \in N, h \neq j, h \neq i, j \neq i} \frac{p_h j \left(i\right)}{p_{hj}} \tag{6}$$

Small worldness

Small world index [5]

$$S = \frac{C/C_{rand}}{L/L_{rand}} \tag{7}$$

C Groupwise modular structure

Table 1: Modular structure identified at a groupwise level

Region	Module
Amygdala_R	Inferior occipital
Calcarine_L	Inferior occipital
Calcarine_R	Inferior occipital
Cuneus_L	Inferior occipital
Cuneus_R	Inferior occipital
Hippocampus_L	Temporal
Hippocampus_R	Temporal
ParaHippocampal_L	Temporal
ParaHippocampal_R	Temporal
Occipital_Inf_R	Temporal
Fusiform_L	Temporal
Fusiform_R	Temporal
Temporal_Mid_R	Temporal
Temporal_Pole_Mid_L	Temporal
Frontal_Sup_L	Frontal/Occipital
Frontal_Sup_R	Frontal/Occipital

Continued on next page

Table 1 – *Continued from previous page*

Region	Module
Frontal_Sup_Orb_L	Frontal/Occipital
Frontal_Sup_Orb_R	Frontal/Occipital
Frontal_Mid_L	Frontal/Occipital
Frontal_Mid_R	Frontal/Occipital
Frontal_Mid_Orb_L	Frontal/Occipital
Frontal_Mid_Orb_R	Frontal/Occipital
Frontal_Inf_Orb_L	Frontal/Occipital
Frontal_Inf_Orb_R	Frontal/Occipital
Olfactory_L	Frontal/Occipital
Olfactory_R	Frontal/Occipital
Rectus_L	Frontal/Occipital
Rectus_R	Frontal/Occipital
Lingual_L	Frontal/Occipital
Lingual_R	Frontal/Occipital
Occipital_Sup_L	Frontal/Occipital
Occipital_Sup_R	Frontal/Occipital
Occipital_Mid_L	Frontal/Occipital
Occipital_Mid_R	Frontal/Occipital
Occipital_Inf_L	Frontal/Occipital
Caudate_L	Frontal/Occipital
Caudate_R	Frontal/Occipital
Temporal_Pole_Sup_L	Frontal/Occipital
Temporal_Pole_Sup_R	Frontal/Occipital
Temporal_Mid_L	Frontal/Occipital
Temporal_Pole_Mid_R	Frontal/Occipital
Temporal_Inf_L	Frontal/Occipital
Temporal_Inf_R	Frontal/Occipital
Medial_Prefront_lower_L	Frontal/Occipital
Medial_Prefront_upper_L	Frontal/Occipital
Medial_Prefront_upper_R	Frontal/Occipital
Rostral_ACC_bilateral	Frontal/Occipital
Pregenual_ACC_bilateral	Frontal/Occipital
Dorsal_ACC_bilateral	Frontal/Occipital
Precentral_L	Parietal premotor
Precentral_R	Parietal premotor
Frontal_Inf_Oper_L	Parietal premotor
Frontal_Inf_Oper_R	Parietal premotor
Frontal_Inf_Tri_L	Parietal premotor
Frontal_Inf_Tri_R	Parietal premotor
Rolandic_Oper_L	Parietal premotor
Rolandic_Oper_R	Parietal premotor
Supp_Motor_Area_L	Parietal premotor
Supp_Motor_Area_R	Parietal premotor
Amygdala_L	Parietal premotor
Postcentral_L	Parietal premotor
Postcentral_R	Parietal premotor
Parietal_Sup_L	Parietal premotor

Continued on next page

Table 1 – *Continued from previous page*

Region	Module
Parietal_Sup_R	Parietal premotor
Parietal_Inf_L	Parietal premotor
Parietal_Inf_R	Parietal premotor
SupraMarginal_L	Parietal premotor
SupraMarginal_R	Parietal premotor
Precuneus_L	Parietal premotor
Paracentral_Lobule_L	Parietal premotor
Paracentral_Lobule_R	Parietal premotor
Putamen_L	Parietal premotor
Putamen_R	Parietal premotor
Pallidum_L	Parietal premotor
Pallidum_R	Parietal premotor
Heschl_L	Parietal premotor
Heschl_R	Parietal premotor
Temporal_Sup_L	Parietal premotor
Temporal_Sup_R	Parietal premotor
Ant_Insula_L	Parietal premotor
Ant_Insula_R	Parietal premotor
Post_Insula_L	Parietal premotor
Post_Insula_R	Parietal premotor
Posterior_MCC_bilateral	Parietal premotor
23d_bilateral	Parietal premotor
Frontal_Mid_Orb_L	Prefrontal
Frontal_Mid_Orb_R	Prefrontal
Angular_L	Prefrontal
Angular_R	Prefrontal
Precuneus_R	Prefrontal
Thalamus_L	Prefrontal
Thalamus_R	Prefrontal
Medial_Prefront_lower_R	Prefrontal
dPCC_bilateral	Prefrontal
vPCC_bilateral	Prefrontal

D Group modularity identification

Simultaneous identification of the optimal modular decompositions is a two phase process. First the landscape for each individual is restricted to only decompositions that maximally overlap the typical decomposition of at least one other member of the group. The typical landscape for an individual is defined by the mode of the landscape for that subject.

The first pass landscape filter can be described as:

$l \leftarrow \emptyset$
for $i = 1 : n$ **do**
  $max_j \leftarrow 0$
  for $j = 1 : m$ **do**
    if $q > max_j$ **then**

$max_j \leftarrow q$
end if
end for
if $max_j \notin l$ **then**
append max_j to l
end if
end for

where l is a list of all surviving decompositions for the subject, q is the goodness of fit between two decompositions, n is the number of decompositions in the landscape and m is the number of subjects in the group.

The second pass recursively finds the best matching pairs of modular decompositions, removes all other possibilities for those subjects and creates a typical decomposition for the combined subjects for which everything is compared to in future comparisons.

References

1. Rubinov M, Sporns O (2011) Weight-conserving characterization of complex functional brain networks. NeuroImage In Press, Corrected Proof: –.

2. Watts DJ, Strogatz SH (1998) Collective dynamics of /'small-world/' networks. Nature 393: 440–442.

3. Latora V, Marchiori M (2001) Efficient behavior of small-world networks. Phys Rev Lett 87: 198701–.

4. Freeman LC (1978) Centrality in social networks conceptual clarification. Social Networks 1: 215–239.

5. Humphries MD, Gurney K (2008) Network 'small-world-ness': a quantitative method for determining canonical network equivalence. PLoS ONE 3: e0002051–e0002051.
